# Diagnostik und Therapie des Typ 1 Diabetes mellitus (Update *2023*)

**DOI:** 10.1007/s00508-023-02182-8

**Published:** 2023-04-20

**Authors:** Monika Lechleitner, Susanne Kaser, Friedrich Hoppichler, Michael Roden, Raimund Weitgasser, Bernhard Ludvik, Peter Fasching, Yvonne Winhofer, Alexandra Kautzky-Willer, Guntram Schernthaner, Rudolf Prager, Thomas C. Wascher, Martin Clodi

**Affiliations:** 1 Avomed – Arbeitskreis für Vorsorgemedizin zbd Gesundheitsförderung in Tirol, Innsbruck, Österreich; 2 Department für Innere Medizin 1, Medizinische Universität Innsbruck, Innsbruck, Österreich; 3 Abteilung für Innere Medizin, Krankenhaus der Barmherzigen Brüder Salzburg, Salzburg, Österreich; 4 Klinik für Endokrinologie und Diabetologie, Medizinische Fakultät, Heinrich-Heine-Universität, Düsseldorf, Deutschland; 5 Institut für Klinische Diabetologie, Deutsches Diabetes-Zentrum (DDZ), Leibniz-Zentrum für Diabetesforschung, Düsseldorf, Deutschland; 6 Deutsches Zentrum für Diabetesforschung (DZD e. V.), München-Neuherberg, Deutschland; 7 Abteilung für Innere Medizin, Privatklinik Wehrle-Diakonissen, Salzburg, Österreich; 8 Universitätsklinik für Innere Medizin I, LKH Salzburg – Universitätsklinikum der Paracelsus Medizinischen Privatuniversität, Salzburg, Österreich; 9 1. Medizinische Abteilung mit Diabetologie, Endokrinologie und Nephrologie, Klinik Landstraße, Wien, Österreich; 10 5. Medizinische Abteilung für Endokrinologie, Rheumatologie und Akutgeriatrie, Wilhelminenspital der Stadt Wien, Wien, Österreich; 11 Klinische Abteilung für Endokrinologie und Stoffwechsel, Universitätsklinik für Innere Medizin III, Medizinische Universität Wien, Wien, Österreich; 12 Gender Medicine Unit, Klinische Abteilung für Endokrinologie und Stoffwechsel, Universitätsklinik für Innere Medizin III, Medizinische Universität Wien, Währinger Gürtel 18–20, 1090 Wien, Österreich; 13 1. Medizinische Abteilung mit Diabetologie, Endokrinologie und Department für Nephrologie, Krankenanstalt Rudolfstiftung, Wien, Österreich; 14 3. Medizinische Abteilung mit Stoffwechselerkrankungen und Nephrologie, Krankenhaus Hietzing, Wien, Österreich; 15 Karl Landsteiner Institut für Stoffwechselerkrankungen und Nephrologie, Wien, Österreich; 16 1. Medizinische Abteilung, Hanusch-Krankenhaus, Wien, Österreich; 17 ICMR – Institute for Cardiovascular and Metabolic Research, Johannes Kepler Universität Linz, Linz, Österreich; 18 Abteilung für Innere Medizin, Konventhospital der Barmherzigen Brüder Linz, Linz, Österreich

**Keywords:** Typ 1 Diabetes mellitus, Autoimmunerkrankungen, Insulintherapie, Type 1 diabetes mellitus, Autoimmune disorders, Insulin therapy

## Abstract

Die Leitlinie nimmt Bezug auf die Diagnostik, einschließlich begleitender Autoimmunerkrankungen, bei Typ 1 Diabetes mellitus, die Insulintherapie und die glykämischen Zielwerte.

## Abstract (en)

This guideline summarizes diagnosis of type 1 diabetes, including accompanying autoimmune disorders, insulin therapy regimens and glycemic target values.

In Bezug auf die Definition werden der Typ 1 und Typ 2 Diabetes mellitus als heterogene Erkrankungen mit unterschiedlicher klinischer Präsentation dargestellt, wobei die Klassifizierung in Typ 1 und Typ 2 Diabetes für die Therapieentscheidung von grundlegender Bedeutung ist [1–3]. Der Typ 1 Diabetes mellitus betrifft rund 5–10 % aller Diabeteserkrankungen. Ein Großteil der Neumanifestationen eines Typ 1 Diabetes tritt im Kindes- und Jugendalter auf („jugendlicher Diabetes mellitus“), grundsätzlich kann sich ein Typ 1 Diabetes aber in jedem Lebensalter manifestieren [1, 3].

Global wurde in den letzten Jahrzehnten ein deutlicher Anstieg der Diabetesinzidenz auf derzeit geschätzte 15 Neudiagnosen pro 100.000 Menschen pro Jahr beobachtet [4].

Der Entwicklung des Typ 1 Diabetes liegt eine zellulär-mediierte Autoimmundestruktion der pankreatischen beta-Zelle zugrunde [1, 2]. Die Autoimmunmarker inkludieren Inselzellantikörper, GAD65 Antikörper, Insulin Antikörper, Antikörper gegenüber Tyrosinphosphatase IA‑2 und IA-2beta, sowie Antikörper gegenüber Zinktransporter 8 (ZnT8) und Tetraspanin‑7 [1, 5, 6].

Der Typ 1 Diabetes wird durch eine fehlende oder inadäquat niedrige Insulinsekretion gemessen mittels C‑Peptid und Vorliegen von einem oder mehreren Autoantikörper definiert. Für eine genetische Prädisposition spricht die starke Assoziation zwischen Typ 1 Diabetes und dem HLA Genotypus, insbesondere DQA und DQB [1, 2].

Das Risiko zur Entwicklung eines Typ 1 Diabetes ist bei Verwandten von Menschen mit Typ 1 Diabetes um das 15–20-fache erhöht und beträgt 6–7 % bei Geschwisterkindern und 25–50 % bei eineiigen Zwillingen [7, 8].

Die Ausprägung der klinischen Symptome eines Typ 1 Diabetes ist variabel, mit Polyurie, Polydipsie, Schwächegefühl, Sehstörungen, Infektneigung und Gewichtsverlust als typischen Anzeichen einer metabolischen Entgleisung bis hin zur diabetischen Ketoazidose. Die diabetische Ketoazidose und begleitende gastrointestinale Beschwerden können vor allem bei Kindern im Rahmen der Erstmanifestation eines Typ 1 Diabetes beobachtet werden [1, 9]. Bei Angehörigen von Menschen mit Typ 1 Diabetes wurde im Rahmen von Studien das Risiko einer Diabetesmanifestation bei positiven Antikörperbefunden untersucht. Aus einer multinationalen Studie bei Kindern geht hervor, dass bei mehr als 2 positiven Antikörpern rund 70 % innerhalb der nächsten 10 Jahre und 84 % innerhalb von 15 Jahren einen Typ 1 Diabetes entwickeln [10].

Neben den klassischen Autoimmunformen des Typ 1 Diabetes wurden in den letzten Jahren – insbesondere in afrikanischen und asiatischen Ethnien – idiopathische Varianten eines Typ 1 Diabetes mellitus beschrieben. Dabei zeigte sich kein serologischer Hinweis auf eine beta-Zell-Autoimmunität, jedoch ein permanenter Insulinmangel mit Ketoazidoseneigung [1, 11].

Eine rasche Entwicklung einer ausgeprägten Insulindefizienz wurde unter Therapie mit Check-Point-Inhibitoren beschrieben [1, 2, 12].

Die vormals als Latent Autoimmune Diabetes in Adults (LADA) bezeichnete Diabetesvariante stellt ein phänotypisches Mischbild dar, das von einem Autoantikörper-positiven Typ 1 Diabetes mit frühzeitiger Insulindefizienz bis zu Formen mit ausreichender beta-Zell-Reserve und Symptomen des metabolischen Syndroms reicht [3, 13–15].

## Therapie des Typ 1 Diabetes mellitus

Eine Grundlage zur erfolgreichen Umsetzung der umfassenden Therapie (Lebensstilmaßnahmen, Glukosekontrolle, Insulintherapie, präventive Maßnahmen betreffend diabetischer Spätkomplikationen) ist die Teilnahme an einer strukturierten Schulung und damit Übernahme der Entscheidungskompetenz in der Therapieumsetzung durch den Patienten/die Patientin selbst (siehe Leitlinie „Diabetesschulung und -beratung bei Erwachsenen mit Diabetes“). Die Insulintherapie stellt bei Typ 1 Diabetes mellitus eine lebensnotwendige Hormonersatztherapie dar. Die umfassende Betreuung des Menschen mit Typ 1 Diabetes sollte grundsätzlich an einem diabetologischen Zentrum bzw. bei einem Arzt mit entsprechender Schwerpunktausbildung für Diabetologie/Endokrinologie erfolgen [1, 2].

### Insulintherapie bei Diabetes mellitus Typ 1

#### Insuline

Zur Insulintherapie werden in Österreich vorwiegend humanes Insulin bzw. Insulinanaloga in einer Konzentration von 100 IE pro ml (U 100) verwendet. Insuline mit höheren Konzentrationen für Patient:innen mit besonders hohem Insulinbedarf sind Insulin Lispro U 200 und Humulin R U 500. Diese höherkonzentrierten Insuline stehen nur in Form von Fertigpens zur Verfügung, um das Risiko von Verwechslungen bei Ampullenwechsel und damit Überdosierungen zu reduzieren.

Die routinemäßige Verabreichung von Insulin erfolgt subkutan mittels Injektionsspritze bzw. Pen oder durch eine Insulinpumpe. Stoffwechselentgleisungen oder Komorbiditäten (perioperative Versorgung) können kurzfristig eine intravenöse Verabreichung von Normalinsulin oder kurzwirksamen Insulinanaloga erforderlich machen. Insuline stehen als kurzwirksame, langwirksame Insuline und Insulinanaloga, sowie als Mischinsuline zur klinischen Anwendung zur Verfügung (Tab. 1 und 2).

**Table 1:** 

Insulin	Wirkungsbeginn (Stunden)	Wirkmaximum (Peak, Stunden)	Wirkdauer (Stunden)
*Normalinsulin*	0,5–1,0	1,5–3,5	7–8
*Kurzwirksame Insulinanaloga* (Lispro, Aspart, Glulisin) *Ultrakurzwirksame Insulinanaloga* (Ultra rapid lispro, Fast-acting aspart)	0,15–0,35 0,1–0,2	1–3	3–5
*NPH-Insulin*	2–4	4–6	8–14
*Langwirksame Insulinanaloga*			
Glargin U 100	2–4	8–12	22–24
Detemir	1–2	4–7	20–24
*Ultralangwirksame Insulinanaloga*		Flache Wirkkurve	
Glargin U 300	2–6	Minimaler Peak	30–36
Degludec	0,5–1,5	Minimaler Peak	> 42

**Table 2:** 

	Sanofi-aventis	Eli Lilly	Novo Nordisk
*Kurzwirksame Insuline*	Insuman® Rapid	Huminsulin® Normal	Actrapid®
*Kurzwirksame Insulinanaloga*	Apidra® (Glulisin)	Humalog® (Lispro)	NovoRapid® (Aspart)
*Ultrakurzwirksame Insulinanaloga*		Lyumjev® (lispro-aabc)	Fiasp® (fast acting aspart)
*Langwirksame Insuline*	Insuman® Basal	Huminsulin® Basal	Insulatard
*Langwirksame Insulinanaloga*	Lantus® (Glargin)	–	Levemir® (Detemir)
*Ultralangwirksame Insulinanaloga*	Lantus® U 300 (Toujeo)		Degludec (Tresiba®)
*Mischinsuline* (NPH-Insulin plus 15 bis 30 % Normalinsulin)	Insuman® Comb 15	Huminsulin® Profil III	Mixtard® 30
	Insuman® Comb 25		
	Insuman® Comb 50		
*Mischinsuline mit Insulinanaloga* NPH-Insulin plus 25 bis 70 % kurzwirksame Insulinanaloga	–	Humalog® Mix 25	NovoMix® 30
		Humalog® Mix 50	NovoMix® 50
			NovoMix® 70
Langwirksames und kurzwirksames Insulinanalogon	–	–	70 % Degludec plus 30 % Aspart (Ryzodeg®)

**Kurzwirksame Insulinanaloga **(Lispro, Aspart, Glulisin) werden seit rund 20 Jahren in der Diabetestherapie eingesetzt [17]. Der gegenüber Normalinsulin raschere Wirkeintritt und die geringere Wirkdauer der kurzwirksamen Insulinanaloga haben die Lebensqualität der Menschen mit Diabetes insofern verbessert, als kein Spritz-Ess-Abstand mehr eingehalten werden muss. In klinischen Studien war die Hypoglykämierate, insbesondere für schwere und nächtliche Hypoglykämien, unter kurzwirksamen Insulinanaloga deutlich geringer als unter Normalinsulin [18–21].

Eine weitere Neuerung stellt die Entwicklung **ultrakurzwirksamer Insulinanaloga **dar. Fast-acting insulin aspart ist ca. 10 min rascher in der Zirkulation wie Insulin aspart und zeigt eine 74 % höhere Insulinwirkung in den ersten 30 min nach Injektion [22]. Klinischen Studien beschreiben für Menschen mit Typ 1 [23] und Typ 2 [24] Diabetes eine stärkere Reduktion des postprandialen Glukosespitzenwertes unter Fast-acting insulin aspart im Vergleich zu Insulin Aspart. Ebenso zeigt Ultra rapid Insulin lispro (lispro-aabc) einen um ca 10 min rascheren Wirkeintritt als Insulin lispro und eine vorteilhafte Beeinflussung der postprandialen Hyperglykämie [25, 26].

Die Entwicklung **langwirksamer Insulinanaloga (Insulin Glargin U 100, Insulin Detemir) **hatte zum Ziel, eine gegenüber NPH-Insulin flachere Wirkkurve und längere Wirkdauer zu erzielen. Langwirksame Insulinanaloga zeigten in klinischen Studien gegenüber NPH-Insulin eine Reduktion vor allem nächtlicher Hypoglykämien [27–29]. Von Vorteil in der klinischen Praxis und Handhabung ist auch das Vorliegen der langwirksamen Insulinanaloga in Form einer klaren Lösung, während bei Applikation von NPH-Insulin eine vorausgehende Suspension des Insulins erforderlich ist.

Zu den sogenannten **ultralangwirksamen Insulinanaloga** zählen Insulin **Glargin U 300** und Insulin **Degludec** [30, 31]. Die lange Wirkdauer und flache Wirkkurve der ultralangwirksamen Insulinanaloga ermöglicht eine Reduktion der Injektionshäufigkeit des basalen Insulins – üblicherweise auf einmal täglich – und größere Flexibilität in der Wahl des Injektionszeitpunktes. In klinischen Studien wurde für die ultralangwirksamen Insuline insgesamt eine gegenüber Insulin Glargin U 100 geringere Hypoglykämierate und geringere Variabilität der Blutzuckerschwankungen beschrieben. Für beide ultralangwirksamen Insulinpräparate liegen Studienreihen zum Einsatz bei Patient:innen mit Typ 1 und Typ 2 Diabetes mellitus vor [32–35].

In Bezug auf die Sicherheit der Insulinanaloga konnten Meta-Analysen unter Einschluss großer Patient:innenpopulationen aus Diabetesregistern keine Zunahme des Tumorrisikos für Insulinanaloga erheben [36, 37].

Zur Verfügung stehen mittlerweile auch Biosimilars für Insulin Glargin und Insulin Lispro [38].

#### Formen der Insulintherapie

Die **funktionelle Insulintherapie** oder Basis-Bolus- Insulintherapie mit ein- bzw. zweimal täglicher Verabreichung eines langwirksamen Basisinsulins/Insulinanalogons und eines kurzwirksamen Insulins/Insulinanalogons prandial bzw. als Korrekturinsulin ist – seit den Publikationen der DCCT-Studie – die Standardform der Insulinbehandlung bei Typ 1 Diabetes [39–41]. Zur Anpassung der Insulintherapie und Übertragung der therapeutischen Entscheidungskompetenz auf die Patient:innen ist dabei die Selbstkontrolle der Glukosewerte eine grundlegende Voraussetzung (siehe Leitlinie „Blutzuckerselbstkontrolle“).

Als eine Variante gilt die **Insulinpumpentherapie** (siehe Leitlinie „Insulinpumpentherapie bei Kindern, Jugendlichen und Erwachsenen“). Eine **konventionelle Form der Insulintherapie** (siehe Leitlinie „Insulintherapie bei Typ 2 Diabetes mellitus“) sollte bei Menschen mit Typ 1 Diabetes nur noch in Ausnahmefällen zum Einsatz kommen.

Die funktionelle Insulintherapie stellt eine dem physiologischen Insulinsekretionsmuster angepasste Form der Insulinsubstitution dar [1, 42]. Bei normaler beta-Zellfunktion erfolgt eine basale Insulinsekretion im Fastenzustand kontinuierlich mit ca. 1,0 E/h und diskontinuierlich entsprechend der Nahrungszufuhr. Die prandiale Freisetzung von Insulin beträgt bei Stoffwechselgesunden für Kohlenhydrate etwa 1,5 E/10 g. Für die Zufuhr von Protein bzw. Fett ist die Insulinfreisetzung wesentlich niedriger und dementsprechend beim Typ 1 Diabetes zur Berechnung des Insulinbedarfs in der täglichen Praxis zu vernachlässigen. Beim Erwachsenen mit Typ 1 Diabetes beträgt bei gewichtserhaltender Ernährung der Anteil des prandialen Insulins somit ca. 50–60 % der Gesamttagesdosis, der Anteil des basalen Insulins ca. 40–50 %. Das basale Insulin ist von entscheidender Bedeutung für die Aufrechterhaltung eines normalen Stoffwechsels im Fastenzustand.

Generell liegt der Insulintagesbedarf bei normalgewichtigen erwachsenen Patient:innen mit neu-diagnostiziertem Typ 1 Diabetes bei insgesamt rund 0,4–1 E/kg Körpergewicht.

Bei der individuellen Anpassung der Insulindosis ist zu berücksichtigen, dass der absolute Insulinbedarf auch von der jeweiligen Insulinsensitivität des Patienten/der Patientin abhängig ist. Die vom Stoffwechselgesunden abgeleiteten Richtwerte für die Insulindosierung beim Patienten mit Diabetes/bei der Patientin mit Diabetes gelten daher nur für den Fall eines absoluten Insulinmangels und einer normalen Insulinsensitivität. Bei einem nur teilweisen Betazellverlust reduziert die verbliebene Insulinrestsekretionsrate den täglichen Insulinbedarf des Patienten/der Patientin, während bei Insulinresistenz der Insulinbedarf erhöht ist. Für einen Großteil der Patient:innen muss die Insulindosierung deshalb individuell angepasst werden, unter Berücksichtigung des Ausmaßes des Insulindefizits, der Insulinsensitivität, der Pharmakokinetik und Pharmakodynamik der Insulinpräparate, der Nahrungszufuhr und der körperlichen Aktivität.

Für die basale Substitution von Insulin stehen NPH-Insuline (Insulatard, Insuman basal, Huminsulin Lilly Basal), langwirksame (Glargin U100, Detemir) und ultralangwirksame Insulinanaloga (Glargin U 300, Insulin Degludec) zur Verfügung. NPH-Insulin wird zur Hälfte morgens und abends verabreicht. Die Injektionsfrequenz kann insbesondere bei den ultralangwirksamen Insulinanaloga auf einmal täglich reduziert werden [1, 2, 33].

Als prandiales Insulin werden Normalinsulin (Humaninsulin) oder kurzwirksame (Aspart, Glulisin, Lispro) oder ultrakurzwirksame Insulinanaloga (Insulin lispro-aabc, Fast-acting Insulin aspart) verabreicht.

Die Dosierung des prandialen Insulins ergibt sich aus der Menge der zugeführten Kohlenhydrate, tageszeitlichen Schwankungen (am Morgen höhere Dosis) sowie der Anpassung an die Glukosezielwerte (Korrekturinsulin). Die Dosis des prandialen Insulins für die zugeführte Menge an Kohlenhydraten beträgt bei Erwachsenen im Durchschnitt 1,0–2,0 IE/BE (1 BE entspricht einer Kohlenhydratmenge von 12 g). Die Korrekturen des prandialen Insulins im Tagesverlauf erfolgen beim Erwachsenen entsprechend der Grundregel, dass 1 IE kurzwirksames Insulin die Blutglukose um 40–50 mg/dL senkt. Eine Anpassung der Dosis an den aktuellen Insulinbedarf ist stets erforderlich (z. B. bei Sportausübung, Infekte, Dehydratation).

Insgesamt angestrebt wird, unter Berücksichtigung der individuellen Gegebenheiten und vor allem des Hypoglykämierisikos, eine normnahe, d. h. den Werten von Menschen ohne Diabetes angenäherte, Kontrolle der Blutglukosewerte [1, 2].

## Blutglukosezielwerte

Die Blutglukosezielwerte im Rahmen der Selbstkontrollen sollten 80–110 mg/dL nüchtern bzw. vor den Mahlzeiten, und vor dem Schlafengehen 110–130 mg/dL betragen [1]. Ideale postprandiale Glukosespitzenwerte (Bestimmung 1–2 h nach Einnahme einer Mahlzeit) liegen unter 180 mg/dL [1]. Diese Glukosewerte entsprechen einem HbA_1c_-Wert von < 7,0 % [1, 2].

Für die kontinuierlichen Glukosemessungen mittels subcutaner Glukosesensoren findet die Zeit im Zielbereich („Time in range“), meist definiert durch Werte zwischen 70–180 mg/dL, [1, 2], als Indikator für die Qualität der Glukosekontrolle Anwendung [1, 2]. Bei einem Anteil von rund 70 % dieser Messungen im Zielkorridorbereich von 70–180 mg/dL ist ein Erreichen des HbA_1c_-Zielwertes von unter 7 % zu erwarten.

Grundlegend für die Zielwertdefinition und Wahl der Therapieform sind die Ergebnisse der DCCT/EDIC-Studie, die bereits 1993 aufzeigen konnte, dass bei Menschen mit Typ 1 Diabetes mit der Senkung des HbA_1c_-Wertes in die Nähe des nicht-diabetischen Normbereichs das Risiko für mikroangiopathische Komplikationen signifikant reduziert wird [39–41]. In der ursprünglichen DCCT-Studie wurde die funktionelle Insulintherapie mit NPH-Insulin und Normalinsulin umgesetzt. Die striktere glykämische Kontrolle war dabei mit einem erhöhten Hypoglykämierisiko assoziiert [39]. Für die in den Folgejahren entwickelten kurz- und langwirksamen Insulinanaloga konnte ein gegenüber NPH- bzw. Normalinsulin reduziertes Hypoglykämierisiko erhoben werden [18, 28, 29].

Die große klinische Bedeutung der Hypoglykämie geht auch aus Auswertungen der EURODIAB IDDM Studie hervor, die nachweisen konnte, dass schwere Hypoglykämien bei Typ 1 Diabetes zu einer Verlängerung der QTc-Zeit und damit zu einem erhöhten Risiko für Herzrhythmusstörungen führen [43].

Nächtliche Glukosekontrollen (ca. 2.00–4.00 Uhr) werden bei Verdacht oder bei bekannter Neigung zu nächtlicher Hypoglykämien empfohlen und sollten darüberhinaus regelmäßig, je nach Stabilität der Stoffwechselkontrolle, alle 4–8 Wochen vorgenommen werden. Als mittlerweile bei vielen Patient:innen eingesetzte Alternative gilt die kontinuierliche Glukosemessung mittels Sensor bzw. Flash-Glukosemessung (siehe Leitlinienabschnitt Glukosesensor).

### Typ 1 Diabetes und Adipositas

Bisher wurden Patient:innen mit Typ 1 Diabetes als schlanke, insulinsensitive Individuen, bei denen der absolute Insulinmangel als Ursache der Hyperglykämie im Vordergrund steht, charakterisiert [1, 2]. Im Zuge des Anstieges der Prävalenz von Übergewicht und Adipositas finden sich auch häufiger übergewichtige und adipöse Patient:innen mit Typ 1 Diabetes [44]. Rezente Untersuchungen zeigen auf, dass sowohl makro- als auch mikrovaskuläre Komplikationen bei Patient:innen mit Typ 1 Diabetes und klinischen Symptomen eines metabolischen Syndroms (von einigen Autoren auch als *Double Diabetes* bezeichnet) signifikant häufiger auftreten [45]. Inwieweit eine Zusatztherapie mit Metformin und Inkretinmimetika für diese Patient:innen von Vorteil sein könnte ist Gegenstand von Diskussionen [46, 47]. So fand sich unter Metformintherapie in der REMOVAL-Studie keine Verbesserung der glykämischen Kontrolle, aber ein günstiger Effekt auf Körpergewicht und LDL-Cholesterin [48].

## Weitere Autoimmunerkrankungen bei Typ 1 Diabetes

Entsprechend Literaturangaben entwickeln bis zu 30 % der Menschen mit Typ 1 Diabetes Autoimmunerkrankungen an weiteren Organsystemen [1, 49–53]. Eine Autoimmunthyroiditis (Morbus Hashimoto oder Basedow) tritt bei 15–30 % der Menschen mit Typ 1 Diabetes auf, eine Autoimmungastritis und/oder perniziöse Anämie bei 5–10 %, eine Zöliakie bei 4–9 %, ein Morbus Addison bei rund 0,5 % und eine Vitiligo bei 2–10 %. Auch das Risiko zur Entwicklung einer Autoimmunhepatitis und einer Myasthenia gravis ist erhöht [1]. Die Diagnostik beruht auf der serologischen Bestimmung organspezifischer Antikörper [54].

Das sogenannte **polyglanduläre Autoimmunsyndrom** I und II ist grundsätzlich mit einem erhöhten Risiko für die Manifestation eines Typ 1 Diabetes assoziiert, rund 20 % der betroffenen Patient:innen entwickeln einen Typ 1 Diabetes [54, 55]. Das Syndrom Typ I stellt eine seltene genetische Erkrankung dar, die auf eine Mutation des Autoimmun-regulatorischen (AIRE) Gens zurückzuführen ist und ein autosomal rezessives Vererbungsmuster aufweist [55]. Die Diagnose erfolgt bei Vorliegen von 2 oder mehreren Teilsymptomen, einschließlich einer mukokutanen Candidiasis, ektodermaler Dysplasien, einer Nebenniereninsuffizienz und/oder eines Hypoparathyroidismus. Die charakteristischen Symptome sind häufig bereits im Kindesalter nachweisbar.

Dem polyglandulären Autoimmunsyndrom II liegt die Assoziation einer endokrinologischen Autoimmunerkrankung mit Einbeziehung weiteren Organsysteme zugrunde. Charakteristika des Typ I Syndroms, insbesondere Mutationen des AIRE Gens, sind nicht nachweisbar. Die Häufigkeit des polyglandulären Autoimmunsyndroms Typ II beträgt 1/20.000 mit einem Überwiegen von Frauen gegenüber Männern im Verhältnis von 3/1. Die höchste Inzidenzrate findet sich im Lebensalter zwischen 20 und 60 Jahren.

### Autoimmunerkrankungen der Schilddrüse

Autoimmunerkrankungen der Schilddrüse führen zum klinischen Bild der Hashimoto Thyroiditis bzw. eines Morbus Basedow. Im Rahmen der Hashimoto Thyroiditis kommt es zum Auftreten von Antikörpern gegen die Thyroid-Peroxidase (TPO) oder Thyreoglobulin, sowie zu einer Erhöhung der TSH Konzentration. Thyroid-Peroxidase Antikörper finden sich bei 15–30 % der erwachsenen Menschen mit Typ 1 Diabetes. Die Prävalenz liegt damit deutlich höher als in der nicht-diabetischen Bevölkerung mit 2–10 % [56]. Rund 50 % der TPO-Antikörper-positiven Patient:innen mit Typ 1 Diabetes zeigen einen Übergang in eine manifeste Schilddrüsenerkrankung. Eine subklinische Hypothyreose findet sich bei 13–20 % der Menschen mit Typ 1 Diabetes, eine subklinische Hyperthyreose bei 6–10 % gegenüber 0,1–2 % in der nicht-diabetischen Bevölkerung [1, 56, 57].

Unter Berücksichtigung diese Datenlage wird in den Leitlinien von Fachgesellschaften, wie der ADA, eine regelmäßige Kontrolle des TSH Wertes als Screening auf eine Schilddrüsenfunktionsstörung empfohlen [1, 58].

### *Zöliakie*

Die Prävalenz der Zöliakie beträgt bei Patient:innen mit Typ 1 Diabetes zwischen 1–8 %, gegenüber 0,5 % in der Allgemeinbevölkerung [1, 59]. Die National Institute for Health and Clinical Excellence (NICE UK) Leitlinien empfehlen ein Screening auf Zöliakie bei der Diagnosestellung eines Typ 1 Diabetes. Entsprechend den Vorgaben von Fachgesellschaften sollte ein Screening auf das Vorliegen einer Glutenenteropathie bei Vorliegen einer entsprechenden klinischen Symptomatik erfolgen [60].

### Autoimmungastritis und Perniziosa

Die Häufigkeit der Autoimmungastritis ist bei Menschen mit Typ 1 Diabetes mit 5–10 % gegenüber der nicht-diabetischen Bevölkerung mit 2–4 % um das 3‑ bis 5‑Fache erhöht [61]. Das Krankheitsbild kann als Teilsymptom des polyglandulären Autoimmunsyndroms auftreten. Eine zumindest einmal jährliche Bestimmung des Blutbilds und des Vitamin B 12 Spiegels ist deshalb bei Patient:innen mit Typ 1 Diabetes empfehlenswert, um Folgekomplikationen zu vermeiden [1, 61].

### Morbus Addison

Antikörper gegen die 21-Hydroxylase finden sich bei 0,7–3 % der Menschen mit Typ 1 Diabetes gegenüber maximal 0,6 % in der nicht-diabetischen Bevölkerung [62]. Entsprechend Literaturangaben beträgt die jährliche Inzidenz eines klinisch manifesten Morbus Addison 20 % [62]. Die klinischen Symptome des Morbus Addison, wie Übelkeit, Erbrechen, Hypotonie, Gewichtsabnahme und Anorexie, können beim Menschen mit Diabetes als Folge einer inadäquaten Behandlung bzw. Therapienebenwirkung missinterpretiert werden. Besondere Beachtung muss die erhöhte Neigung zu Hypoglykämien finden.

### Vitiligo

Der Vitiligo liegt eine Autoimmunerkrankung mit der Ausbildung von Antikörpern gegenüber Melanozyten zugrunde. Bei Vitiligo ist das Risiko für die Manifestation von weiteren Autoimmunerkrankungen erhöht und beträgt rund 10 % für das Auftreten eines Typ 1 Diabetes mellitus [63].

### Screeningempfehlungen

Unter Bezugnahme auf die Literatur und internationale Leitlinienempfehlungen:

- TSH-Kontrolle jedes 2. Jahr bei asymptomatischen und Antikörper negativen Patient:innen, ansonsten häufiger [1].
- Zöliakie Screening bei Erstdiagnose [1, 2]. Berücksichtigt werden muss dabei das bei Menschen mit Typ 1 Diabetes erhöhte Risiko für einen IgA-Mangel mit falsch negativen serologischen Testergebnissen [64, 65].
- Klinisches Bild mit Verdacht auf Morbus Addison, Hyponatriämie und/oder Hyperkaliämie: bei positivem Nebennierenrinden Antikörperbefund regelmäßige ACTH-Verlaufskontrolle und gegebenenfalls weitere Abklärung [1].
